# Spermine Synthase at the Polyamine–Redox Interface: Mechanistic Evidence from Snyder–Robinson Syndrome and Cancer

**DOI:** 10.3390/antiox15101248

**Published:** 2026-09-28

**Authors:** Ziling Lin, Yuning Lin, Mingle Li, Songye Zhan, Qian Xu, Feiyuan Yu

**Affiliations:** 1 Department of Cell Biology and Genetics, Shantou University Medical College, Shantou 515041, China; 22zllin@stu.edu.cn (Z.L.); 23ynlin@stu.edu.cn (Y.L.); 25mlli@stu.edu.cn (M.L.); 23syzhan@stu.edu.cn (S.Z.); 2 International Science and Technology Cooperation Base for Molecular Medicine, Department of Pathology, Shantou University Medical College, Shantou 515041, China

**Keywords:** spermine synthase, polyamine metabolism, antioxidant defense, spermidine/spermine ratio, oxidative stress, ferroptosis, cancer, Snyder–Robinson syndrome

## Abstract

Polyamines are essential metabolites whose biological effects depend on the balance among putrescine, spermidine, and spermine. Spermine synthase (SMS) catalyzes the terminal step of mammalian spermine biosynthesis, simultaneously consuming spermidine and producing spermine. We use this reaction as the organizing framework of the review: SMS changes redox biology indirectly by redistributing spermidine and spermine and by altering the substrate available for oxidative catabolism. In Snyder–Robinson syndrome (SRS), severe SMS deficiency causes spermine depletion, spermidine accumulation, oxidative and organelle stress, and multisystem disease. In cancer, SMS-dependent remodeling can support or restrain tumor growth according to the resulting polyamine profile and downstream metabolic context. Intact spermine can scavenge reactive species and chelate Fe^2+^, whereas SMOX- and SSAT–PAOX-mediated oxidation generates H_2_O_2_ and reactive aldehydes; these opposing routes help explain divergent effects on lipid peroxidation and ferroptosis. This review focuses on evidence that directly connects SMS perturbation or altered spermidine/spermine balance with redox-relevant outcomes in SRS and cancer, distinguishes causal studies from metabolite-level associations, and defines the present limits of therapeutic translation. Together, these findings identify the SMS–polyamine axis as a regulator of the balance between antioxidant protection and oxidase-driven oxidative stress.

## 1. Introduction

Polyamines—putrescine, spermidine, and spermine—are small polycationic metabolites required for macromolecular organization, translation, growth, organelle function, and stress adaptation [1,2,3,4]. Their concentrations are maintained through tightly coupled biosynthesis, interconversion, catabolism, transport, and export [5,6]. Spermine synthase (SMS) catalyzes the terminal aminopropyl-transfer reaction of mammalian spermine biosynthesis by converting spermidine to spermine. Because this reaction consumes one bioactive polyamine while producing another, altered SMS activity changes absolute polyamine concentrations and the spermidine/spermine ratio rather than simply altering total polyamine abundance. Severe loss of activity causes the X-linked Snyder–Robinson syndrome (SRS), whereas context-dependent changes in the SMS–spermine axis have been implicated in cancer and selected non-neoplastic disorders.

SMS does not directly catalyze redox reactions; instead, it influences antioxidant protection by controlling the spermidine and spermine pools. By redistributing these pools, SMS changes the availability of intact polyamines for antioxidant and iron-buffering functions and the supply of substrates for oxidative catabolism. Intact spermine can scavenge reactive species and chelate Fe^2+^, whereas SMOX- or SSAT–PAOX-mediated oxidation generates H_2_O_2_ and reactive aldehydes [5,6,7,8,9]. The net effect therefore depends on polyamine composition, catabolic flux, subcellular distribution, iron availability, mitochondrial state, and endogenous antioxidant capacity.

Accordingly, this review is organized around two disease settings in which the SMS–polyamine–redox relationship is supported by direct mechanistic evidence: SRS and cancer. We first define the enzymatic consequences of changing SMS activity and then examine how the resulting polyamine profile affects oxidative stress, organelle function, lipid peroxidation, and ferroptosis. Throughout the review, we distinguish direct SMS perturbation, metabolite-level intervention without SMS manipulation, and transcriptomic or clinical association. Therapeutic implications are considered only in relation to these evidence levels. Understanding this balance is important for predicting when SMS perturbation will preserve antioxidant protection, increase oxidative injury, or alter ferroptosis sensitivity.

## 2. Polyamines and Polyamine Metabolism

### 2.1. Polyamine Species and Redox-Relevant Functions

Putrescine, spermidine, and spermine are positively charged metabolites that bind nucleic acids and proteins and support translation, growth, and organelle function [1,2,3,4]. Because they differ in charge, compartmentalization, and metabolic fate, their effects cannot be inferred from total polyamine abundance.

Spermidine and spermine have overlapping but non-identical functions. Intact spermine can scavenge reactive species [7,8] and chelate Fe^2+^, thereby limiting lipid peroxidation and ferroptosis [9]. The same polyamine pool can also feed SMOX- or SSAT–PAOX-mediated catabolism, which generates H_2_O_2_ and reactive aldehydes [5,6]. The net redox outcome therefore depends on polyamine species, compartmentalization, iron availability, and catabolic flux.

### 2.2. Polyamine Balance as the Relevant Unit of Analysis

SMS changes two metabolite pools at the same time: reduced activity raises spermidine relative to spermine, whereas increased activity favors spermine, subject to upstream synthesis, transport, and catabolism. Absolute spermine and spermidine concentrations and a clearly oriented ratio are therefore more informative than total polyamine content alone.

Disease studies illustrate this principle. SRS combines a high spermidine/spermine ratio with abnormal catabolism and organelle stress [10,11]. In cancer, SMS perturbation can alter cell survival and ferroptosis response, but the direction of effect depends on the residual polyamine profile and downstream oxidative metabolism [9,12,13,14,15].

### 2.3. Polyamine Metabolic Network and the Central Role of SMS

Polyamine homeostasis is governed by a tightly regulated metabolic network that integrates biosynthesis, interconversion, and catabolism. In mammalian cells, polyamine biosynthesis is initiated by the conversion of L-ornithine to putrescine (PUT), a reaction catalyzed by the rate-limiting enzyme ornithine decarboxylase (ODC). Concurrently, another rate-limiting enzyme, S-adenosylmethionine decarboxylase (AdoMetDC), catalyzes the conversion of S-adenosylmethionine (SAM) to decarboxylated SAM (dcSAM). Spermidine synthase (SRM) catalyzes the transfer of an aminopropyl group from dcSAM to putrescine to generate spermidine, which is subsequently converted to spermine by SMS [5].

Mammalian polyamine catabolism primarily proceeds through two distinct pathways. The first pathway involves the acetylation of spermine or spermidine by spermidine/spermine N^1^-acetyltransferase (SSAT) to form N^1^-acetylspermine or N^1^-acetylspermidine, which are subsequently oxidized to spermidine or PUT, respectively, by N^1^-acetylpolyamine oxidase (PAOX, historically also referred to as APAO). Alternatively, spermine can be directly oxidized to spermidine by spermine oxidase (SMOX) without prior acetylation. Both catabolic routes generate hydrogen peroxide (H_2_O_2_) and reactive aldehydes: PAOX produces 3-acetamidopropanal, whereas SMOX produces 3-aminopropanal [5,6] (Figure 1).

Within this network, SMS is the sole mammalian enzyme responsible for spermine synthesis. It therefore controls the relative pools of spermidine and spermine and, indirectly, the balance between the antioxidant functions of intact polyamines and the pro-oxidant products of polyamine oxidation. This metabolic position, rather than SMS expression alone, is the basis for interpreting its disease effects.

## 3. Spermine Synthase: Structure, Function, and Regulation

### 3.1. Gene and Protein Overview of SMS

SMS is the terminal and non-redundant aminopropyltransferase in mammalian spermine biosynthesis. Biochemical purification established that human spermine synthase is distinct from spermidine synthase [16], and subsequent molecular studies cloned the human *SMS* cDNA and characterized the *SMS*-containing region on the X chromosome [17,18]. Current annotation places the human *SMS* gene at Xp22.11 (Figure 2A) and identifies pathogenic variants in this X-linked gene as the cause of SRS [19]. The experimentally determined structure of human SMS provides a framework for interpreting its catalytic residues and disease-associated variants [20].

### 3.2. Structure and Catalytic Mechanism of Spermine Synthase

X-ray crystallography established that human SMS forms a homodimer (Figure 2B) and that each monomer contains an N-terminal domain (residues 1–117) that shares structural similarity with S-adenosylmethionine decarboxylase (AdoMetDC), a central domain comprising four antiparallel β-strands (residues 138–172), and a C-terminal catalytic domain (residues 173–366) [20].

SMS transfers an aminopropyl group from decarboxylated S-adenosylmethionine (dcSAM, also known as S-adenosyl-3-(methylsulfanyl)propylamine) to spermidine, producing spermine and 5′-S-methylthioadenosine (MTA). The active site lies between the central and C-terminal domains and includes conserved acidic residues Asp201, Asp276, and Glu353. Site-directed mutagenesis directly demonstrated that Asp201 and Asp276 are critical for catalysis [20].

Although the N-terminal domain does not directly participate in catalysis, it is critical for protein stability and dimer formation [20], both of which are essential for enzymatic activity. Mutations in this domain have been implicated in Snyder–Robinson syndrome (SRS), an X-linked intellectual disability syndrome caused by defects in the *SMS* gene. A structural study evaluated five SRS-associated missense variants of spermine synthase—p.Met35Arg (M35R), p.Gly56Ser (G56S), p.Phe58Leu (F58L), p.Gly67Glu (G67E), and p.Pro112Leu (P112L). Experimental analysis of patient-derived cells showed that M35R, G56S, and G67E were associated with the largest reductions in mutant SMS protein abundance, whereas computational modeling predicted that G56S, F58L, G67E, and P112L reduced dimerization affinity [21].

These findings suggest that structural destabilization and impaired dimer formation may contribute to reduced SMS activity in several SRS-associated missense variants. However, the molecular consequences vary among variants and may involve impaired folding, reduced protein stability, altered dimerization, or catalytic impairment. The effects of individual *SMS* variants should therefore be interpreted on a variant-specific basis rather than attributed uniformly to defective dimerization.

### 3.3. SMS as a Metabolic Control Point in Polyamine Homeostasis

SMS catalyzes the terminal step of mammalian spermine biosynthesis by converting spermidine to spermine. Because this reaction simultaneously consumes spermidine and produces spermine, changes in SMS activity redistribute intracellular polyamine pools rather than simply altering total polyamine abundance. SMS deficiency typically increases the spermidine/spermine ratio, whereas increased SMS expression or stability can favor spermine accumulation; however, the magnitude of these changes also depends on upstream polyamine synthesis, transport, export, and catabolism [5,10,11,22].

This redistribution is biologically relevant because spermidine and spermine have overlapping but non-identical cellular functions. Experimental studies link altered SMS activity to proliferation, mitochondrial function, and cellular stress responses [10,11,12,22]. Within the broader pathway, SMS is coupled metabolically—not through a demonstrated direct protein interaction—to SSAT, PAOX, and SMOX. The consequences of SMS dysregulation therefore depend on the resulting polyamine profile and downstream catabolic activity.

### 3.4. Polyamine Balance in Oxidative and Organelle Stress

SMS controls spermidine-to-spermine conversion and therefore affects both the cellular pool of polyamines and the substrates available for protective functions or oxidative catabolism. Intact spermine can scavenge reactive species and protect cellular macromolecules from oxidative damage [7,8]. It can also chelate Fe^2+^, thereby reducing labile iron availability, lipid peroxidation, and ferroptosis [9]. Recent work showed that spermidine reduced probe-accessible Fe^2+^ in a cell-free assay. In K562 cells, spermidine supplementation rescued the ferroptosis sensitivity caused by pharmacological polyamine depletion, whereas SMS-knockout cells depleted of spermine remained resistant to GPX4 inhibition, consistent with a compensatory anti-ferroptotic role for spermidine in this setting [14]. In contrast, oxidative polyamine catabolism produces reactive metabolites. SMOX directly oxidizes spermine, whereas PAOX oxidizes N^1^-acetylated polyamines; both reactions generate H_2_O_2_ and reactive aldehydes [5,6]. Under ferroptosis-inducing conditions, PAOX- and SMOX-dependent polyamine oxidation can promote lipid peroxidation and ferroptotic cell death in cancer cells [15]. The redox effects of altered SMS activity depend on polyamine composition and catabolic flux, as well as cellular compartmentalization, mitochondrial function, iron availability, and antioxidant capacity.

Experimental models of SMS deficiency have reported different oxidative and organelle stress phenotypes. In *dSms*-deficient *Drosophila,* increased spermidine catabolism was accompanied by elevated aldehyde and ROS levels, lysosomal dysfunction, impaired autophagy–lysosome flux, and mitochondrial abnormalities [10]. Related lysosomal, autophagic, or mitochondrial defects were also identified in SRS patient-derived fibroblasts, bone marrow stromal cells, and lymphoblastoid cells [10]. G56S mice had reduced brain spermine levels and lower cortical expression of several OXPHOS-related genes. Primary fibroblasts from G56S mice and CRISPR-generated *Sms*-knockout murine hippocampal cells also showed impaired mitochondrial respiration [11]. Gy/Y-derived skin fibroblasts, however, were more resistant to oxidative stress but more sensitive to UV-C irradiation than wild-type X/Y fibroblasts [23]. These findings are not directly contradictory because they differ in model system, cell type, stressor, and experimental endpoint. Basal disease-associated oxidative stress should also be distinguished from cell survival following an acute external challenge. Thus, SMS deficiency cannot be described as uniformly increasing or decreasing cellular resistance to oxidative stress.

Cellular antioxidant capacity may modify the consequences of SMS deficiency. Overexpression of glutathione S-transferase E1 reduced ROS accumulation and partially rescued mitochondrial, neurological, and survival phenotypes in *dSms*-deficient *Drosophila* [10]. Activation of *SAT1*, which encodes SSAT, has also been shown to increase glutamine-derived GSH synthesis in lung cancer cells, linking polyamine catabolism to GSH metabolism [24]. Whether SMS-dependent changes in polyamine composition also affect nuclear factor erythroid 2-related factor 2 (NRF2) or thioredoxin signaling remains unknown.

Evidence from cancer models further links spermine availability to organelle stress and ferroptosis. In Newcastle disease virus-infected tumor cells, exogenous spermine attenuated Golgi stress and Golgiphagy, reduced ROS accumulation and lipid peroxidation, and suppressed ferroptosis [25]. Because this study used exogenous spermine in a viral-infection model, its findings support a context-specific protective effect of spermine rather than a general role of endogenous SMS in ferroptosis. In HCC cell models, *SMS* knockout or N-(3-aminopropyl)cyclohexylamine (APCHA)-mediated SMS inhibition reduced intracellular spermine levels, increased H_2_O_2_, acrolein, and lipid ROS, and sensitized cells to RSL3- or erastin-induced ferroptosis, indicating that SMS-dependent spermine availability contributes to ferroptosis resistance [9]. The contrasting responses of HCC and K562 cells suggest that the ferroptotic consequences of SMS loss are influenced by cellular context and the residual polyamine pool, including the extent to which spermidine can compensate for spermine depletion [9,14].

### 3.5. Regulation of SMS Expression and Protein Stability

Current evidence identifies three context-specific routes that increase SMS abundance or spermine production: FOXP3-dependent transcription in HCC regulatory T cells [26], METTL3–IGF2BP3-mediated stabilization of *SMS* mRNA in pancreatic cancer [22], and Serpin B9-mediated protection of SMS from lysosomal degradation [27]. These mechanisms define upstream regulation of the SMS–spermine axis, but they do not, by themselves, establish a general SMS-dependent redox program.

## 4. Mechanistic Evidence in SRS and Cancer

### 4.1. SMS and Snyder–Robinson Syndrome

SRS provides the clearest disease model of severe SMS loss. Pathogenic *SMS* variants reduce or abolish enzyme activity, producing spermine depletion, spermidine accumulation, and an elevated spermidine/spermine ratio [19]. The clinical phenotype includes neurodevelopmental, skeletal, and seizure manifestations [28,29,30,31,32,33,34,35,36,37,38,39,40,41,42], but its relevance to the present framework lies in how this altered polyamine profile changes catabolic load and organelle function.

Mechanistic studies indicate that SMS deficiency disrupts organelle homeostasis in addition to reducing spermine availability. In *Drosophila* models and patient-derived cells, excessive spermidine catabolism generates toxic metabolites associated with lysosomal dysfunction, impaired autophagy–lysosome flux, mitochondrial abnormalities, and oxidative stress [10]. The G56S mouse model similarly showed elevated spermidine and reduced spermine levels, resulting in an increased spermidine/spermine ratio, together with growth and skeletal abnormalities, neurobehavioral defects, and impaired mitochondrial oxidative phosphorylation; reduced respiratory capacity was also demonstrated in G56S fibroblasts and *Sms*-knockout murine hippocampal cells [11]. Together, these findings link the abnormal polyamine profile in SRS to organelle dysfunction, while stress responses may vary among tissues and experimental models.

Together, the SRS evidence supports a causal sequence from loss of SMS activity to polyamine imbalance, excessive spermidine catabolism, and mitochondrial and lysosomal stress. Clinical severity and stress responses still vary by variant, tissue, and residual activity; therefore, neither the spermidine/spermine ratio nor oxidative stress alone explains the full phenotype.

A detailed compilation of reported *SMS* variants and clinical features is provided in Supplementary Table S1.

### 4.2. SMS and Cancer

SMS has context-dependent effects in cancer because altering its activity changes both spermine production and spermidine consumption. The strongest mechanistic studies therefore combine SMS perturbation with polyamine measurements and relevant downstream readouts. The discussion below separates tumor-cell mechanisms from redox and ferroptosis outcomes, while transcriptomic associations are treated as supportive evidence.

Exploratory GEPIA2 [43] expression and survival analyses are provided in Supplementary Figure S1 and are interpreted as hypothesis-generating rather than as evidence of SMS activity or pathway flux.

Taken together, current evidence supports context-specific roles for SMS in selected tumors, but the strength of evidence varies from direct genetic or pharmacological intervention to transcriptomic association alone. *SMS* mRNA expression should therefore not be treated as a surrogate for enzyme activity or pathway flux. Functional conclusions require direct pathway perturbation together with measurements of SMS protein or activity, absolute spermidine and spermine concentrations, their explicitly oriented ratio, polyamine transport, and relevant downstream readouts.

#### 4.2.1. SMS and Polyamine-Dependent Metabolic Fitness

At the metabolic level, SMS controls both spermine production and spermidine consumption, thereby shaping intracellular polyamine composition and the spermidine/spermine ratio. The biological consequences of SMS dysregulation may therefore reflect spermine depletion, spermidine accumulation, or a combination of both.

In head and neck squamous cell carcinoma (HNSCC), spermine accumulated in tumor tissue and tumor-derived exosomes, SMS expression was elevated, and SMS knockdown reduced proliferation, colony formation, and migration [13]. Because downstream signaling and paired spermidine/spermine measurements were not reported, this study supports an SMS-dependent growth phenotype but does not define the responsible redox mechanism.

#### 4.2.2. SMS and Oncogenic Signaling Networks

In pancreatic cancer, METTL3-mediated m^6^A modification and IGF2BP3 binding stabilize *SMS* mRNA. SMS overexpression increased spermine, AKT phosphorylation, EMT-associated changes, xenograft growth, and experimental metastasis, whereas SMS depletion produced the opposite effects and increased spermidine [22]. This directly links regulated SMS expression to polyamine redistribution and AKT–EMT signaling, although redox endpoints were not assessed.

In colorectal cancer, SMS disruption caused pronounced spermidine accumulation, reduced spermine and putrescine, and activated the FOXO3a–BIM apoptotic axis; restoring SMS, but not adding spermine or putrescine alone, corrected the growth defect. MYC-dependent miR-19a/19b limited BIM induction, and combined SMS–MYC inhibition produced greater apoptosis and tumor regression [12]. These rescue experiments show that altered polyamine composition, rather than spermine depletion alone, is mechanistically important.

#### 4.2.3. Redox Adaptation, Ferroptosis, and Context-Dependent Tumor Effects

Although several functional studies support tumor-promoting effects of increased SMS activity or spermine production, neither SMS expression nor spermine abundance has a uniform biological effect across tumor types. The outcome depends on the resulting polyamine composition, tissue-specific signaling programs, downstream polyamine catabolism, and whether the observed effect is mediated by SMS itself or by spermine as its downstream product. Accordingly, SMS-dependent remodeling can shift both antioxidant buffering by intact polyamines and the production of pro-oxidant catabolites, thereby changing the redox threshold for tumor-cell survival.

Within polyamine metabolism, the redox outcome depends strongly on the metabolic fate of spermine. In *Helicobacter pylori*-associated gastric carcinogenesis, SMOX-mediated spermine oxidation generates H_2_O_2_, promotes oxidative DNA damage, and contributes to carcinogenic progression [44]. Conversely, recent work in hepatocellular carcinoma identified spermine as an endogenous Fe^2+^ chelator that limits lipid peroxidation and ferroptosis. In HCC cells, *SMS* knockout or inhibition with APCHA reduced spermine levels, increased intracellular H_2_O_2_, acrolein, and lipid ROS levels, and sensitized cells to RSL3- or erastin-induced ferroptosis. In vivo, genetic or pharmacological inhibition of the upstream ALDH18A1-dependent spermine pathway increased lipid peroxidation and impaired hepatocarcinogenesis; these effects were reversed by spermine or liproxstatin-1 [9]. By contrast, *SMS*-knockout K562 cells lacked spermine but accumulated spermidine and remained resistant to GPX4 inhibition, suggesting that spermidine was sufficient to maintain ferroptosis resistance in this setting [14]. Under ferroptosis-inducing conditions, however, PAOX- and SMOX-dependent polyamine oxidation can generate H_2_O_2_ and amplify lipid peroxidation [15]. Together, these findings indicate that ferroptosis sensitivity following SMS disruption depends on the residual polyamine profile and the extent of oxidative polyamine catabolism. By controlling spermine production, SMS helps set the balance between these routes, while the net redox outcome also depends on iron availability, PAOX and SMOX activity, and cellular antioxidant capacity, including the GSH–GPX4 system.

Taken together, cancer studies do not support a single redox consequence of SMS loss. In HCC, spermine depletion increased lipid peroxidation and ferroptosis, whereas in K562 cells, accumulated spermidine preserved ferroptosis resistance. The relevant predictor is therefore the residual polyamine profile together with iron availability, PAOX/SMOX activity, and antioxidant capacity, not SMS expression or the spermidine/spermine ratio in isolation.

Figure 3 summarizes the central SMS–polyamine–redox framework. Table 1 summarizes the evidence retained in the main text and distinguishes direct SMS manipulation from metabolite- or oxidase-level experiments. This evidence hierarchy helps separate causal pathway studies from associations and shows why uniform SMS inhibition cannot be assumed across tumors.

## 5. Therapeutic Implications and Evidence Gaps

The therapeutic implication of this redox framework is that polyamine-directed interventions should be selected according to a defined metabolic state rather than according to total polyamine abundance alone. In SMS-dependent cancers, inhibition may be useful when reduced spermine or altered polyamine composition creates a defined vulnerability; in SRS, the aim is to restore spermine or reduce harmful spermidine accumulation and catabolism. The evidence is therefore organized below by disease mechanism rather than as a broad list of polyamine-related applications. The redox effects of these strategies should therefore be evaluated through concurrent measurements of absolute spermidine and spermine concentrations, PAOX/SMOX activity, labile iron, H_2_O_2_ or reactive aldehydes, lipid peroxidation, and GSH–GPX4 status.

### 5.1. Targeting SMS and Polyamine Biosynthesis in Cancer

The spermine synthase inhibitor N-(3-aminopropyl)cyclohexylamine (APCHA) reduced spermine with compensatory spermidine accumulation in HTC hepatoma cells, with little change in total polyamine content or cell growth [45]. Combining APCHA with the ODC inhibitor α-difluoromethylornithine (DFMO) produced greater growth inhibition than DFMO alone in FM3A and P388 models [46]. These experiments show that SMS inhibition redistributes polyamine pools and that simultaneous restriction of upstream synthesis may limit compensation.

More recent studies identify context-specific vulnerabilities. SMS loss inhibited colorectal cancer growth and increased apoptosis induced by MYC inhibition [12], while *SMS* knockout or APCHA treatment sensitized HCC cells to ferroptosis [9]. These findings support mechanism-guided combinations in tumors in which SMS dependence and the resulting polyamine profile are measured directly.

α-Difluoromethylornithine (DFMO; eflornithine) irreversibly inhibits ODC and reduces upstream polyamine biosynthesis. In 2023, the US Food and Drug Administration (FDA) approved eflornithine to reduce relapse risk in adult and pediatric patients with high-risk neuroblastoma who had achieved at least a partial response to prior multiagent, multimodality therapy, including anti-GD2 immunotherapy [47]. Thus, the approval of eflornithine provides a clinical precedent for targeting polyamine biosynthesis but does not constitute clinical validation of direct SMS inhibition.

### 5.2. Restoring Polyamine Homeostasis in SRS

In SRS, treatment must address spermine deficiency, spermidine excess, or toxic downstream catabolism rather than inhibit SMS indiscriminately. In patient-derived cells and *Drosophila*, a stable spermine analogue and a redox-sensitive spermine prodrug restored spermine-related functions [48,49]. DFMO lowered the abnormal spermidine/spermine ratio when residual SMS activity was present [50], and phenylbutyrate reduced pathological catabolism and improved mitochondrial and autolysosomal phenotypes [51]. Candidate pharmacological chaperones and adeno-associated virus (AAV)-mediated SMS delivery are also under study [52,53].

These approaches remain preclinical. Their rational evaluation requires stratification by SMS variant, residual enzyme activity, tissue distribution, and baseline spermidine and spermine concentrations.

## 6. Conclusions

SMS is best understood as a regulator of polyamine composition rather than as a simple source of spermine. By consuming spermidine and producing spermine, the enzyme changes the relative availability of intact polyamines and the substrate pool for oxidative catabolism. This framework links SMS loss in SRS to spermine depletion, spermidine accumulation, abnormal catabolism, and mitochondrial and lysosomal stress. It also explains why cancer studies yield different outcomes: in HCC, loss of spermine can increase lipid peroxidation and ferroptosis, whereas in K562 cells, accumulated spermidine can preserve ferroptosis resistance.

Accordingly, SMS expression, spermine abundance, and the spermidine/spermine ratio should not be used interchangeably. Mechanistic studies should report SMS activity together with absolute spermidine and spermine concentrations, PAOX/SMOX activity, iron availability, and relevant antioxidant and organelle readouts. Therapeutic inhibition is most plausible where direct SMS dependence and a predictable redox vulnerability have been demonstrated; SRS requires the opposite strategy of restoring spermine function or reducing harmful spermidine accumulation and catabolism. Keeping these disease-specific directions separate provides a clearer basis for further preclinical work. Overall, the translational relevance of the SMS–polyamine axis lies in its capacity to shift the balance between antioxidant protection and pro-oxidant catabolism rather than in a uniformly antioxidant or pro-oxidant function.

## Figures and Tables

**Figure 1:**
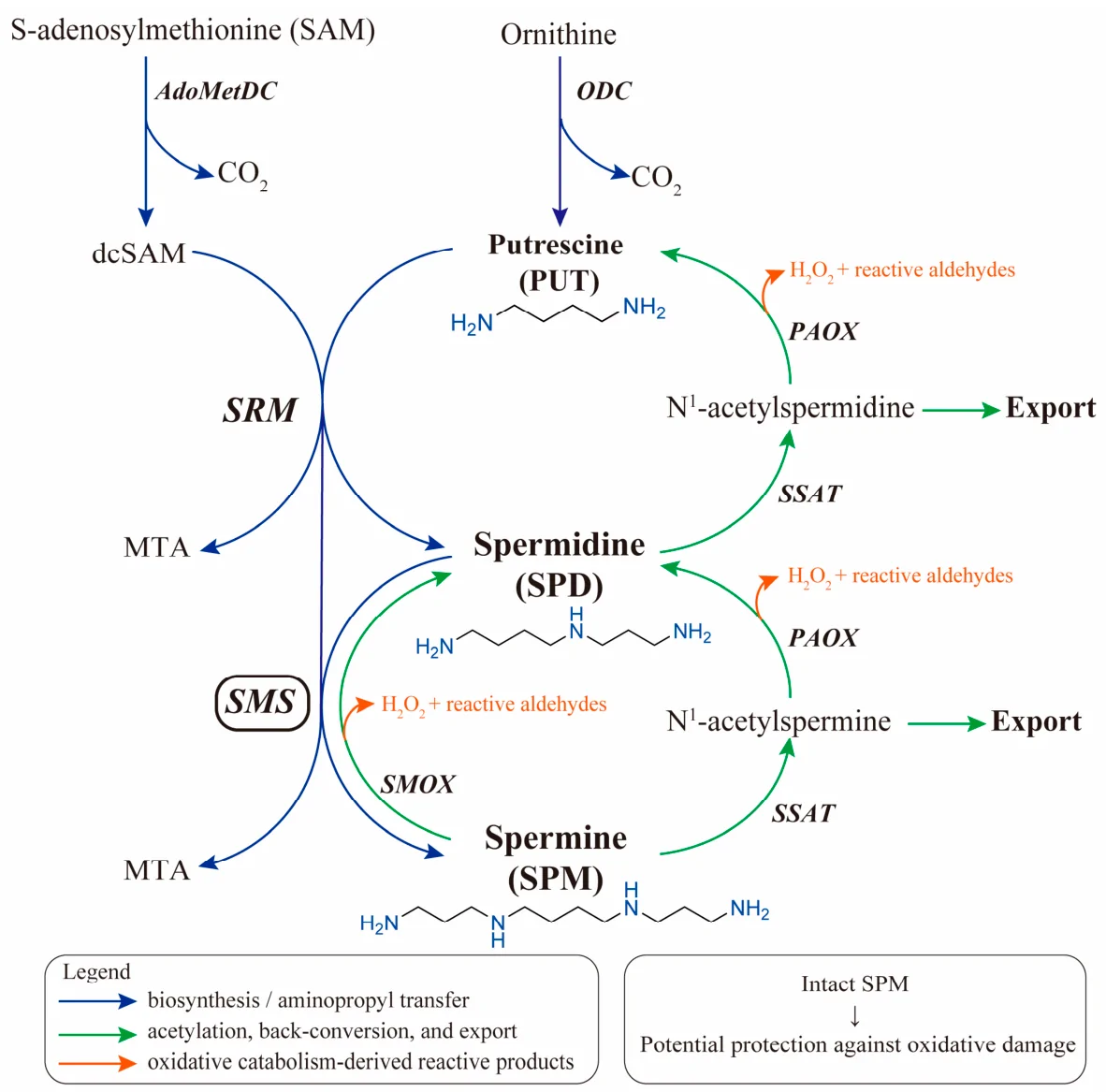
Polyamine metabolic pathway and major redox-relevant reactions. Ornithine decarboxylase (ODC) converts ornithine to putrescine (PUT), whereas S-adenosylmethionine decarboxylase (AdoMetDC) generates decarboxylated S-adenosylmethionine (dcSAM) from S-adenosylmethionine (SAM). Sequential aminopropyl-transfer reactions catalyzed by spermidine synthase (SRM) and spermine synthase (SMS) generate spermidine (SPD) and spermine (SPM), respectively, with 5′-methylthioadenosine (MTA) released during both reactions. In the catabolic pathway, spermidine/spermine N^1^-acetyltransferase (SSAT) acetylates SPD and SPM to N^1^-acetylspermidine and N^1^-acetylspermine, respectively. PAOX subsequently oxidizes these acetylated polyamines to PUT and SPD, respectively, generating H_2_O_2_ and 3-acetamidopropanal. Spermine oxidase (SMOX) directly oxidizes SPM to SPD, generating H_2_O_2_ and 3-aminopropanal. Intact SPM may protect cellular macromolecules and organelles from oxidative damage in a context-dependent manner. The balance between spermine synthesis, utilization, and oxidative catabolism contributes to polyamine homeostasis and shapes cellular responses to oxidative and metabolic stress. Polyamine structures were drawn in ChemDraw (version 23.1.1) based on ChEBI records (CHEBI:17148, CHEBI:16610, and CHEBI:15746); all other graphical elements were created in Adobe Illustrator (version 23.1).

**Figure 2:**
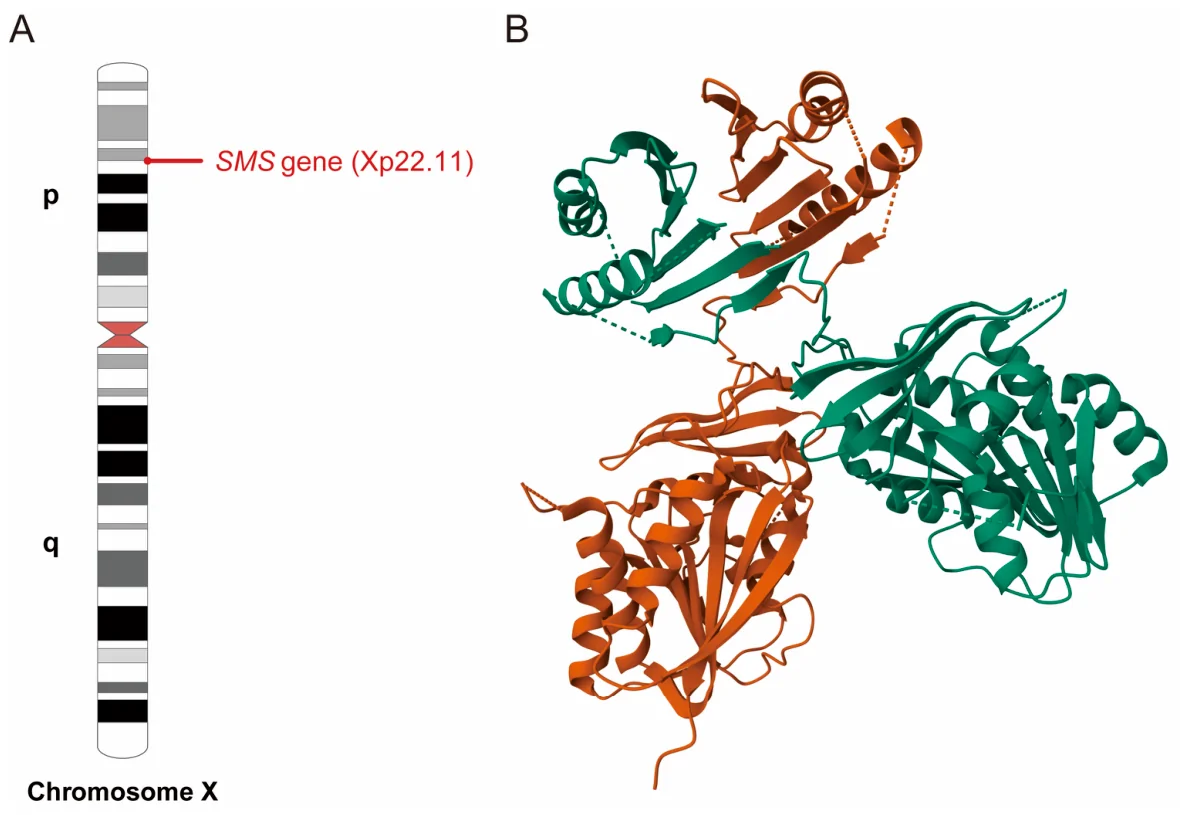
Chromosomal localization of the *SMS* gene and experimentally determined structure of human spermine synthase. (**A**) Location of the *SMS* gene at Xp22.11 on the human X chromosome. (**B**) Three-dimensional structure of human SMS (green and brown distinguish the two subunits of the SMS homodimer). The X chromosome ideogram was generated using R/RCircos (hg38 cytoband). SMS structure is based on PDB ID 3C6K.

**Figure 3:**
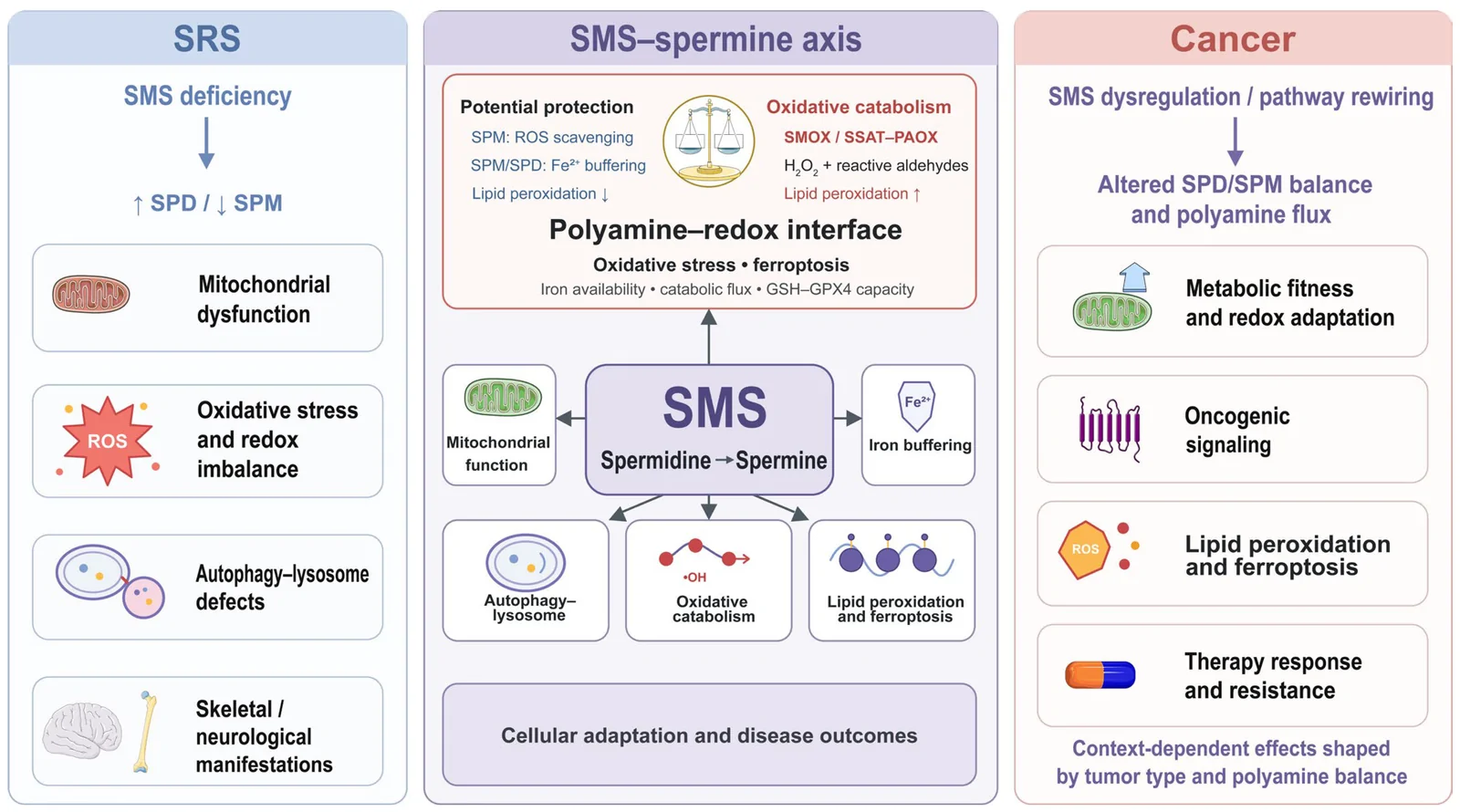
Central framework linking SMS-dependent polyamine remodeling to redox outcomes in SRS and cancer. SMS converts spermidine (SPD) to spermine (SPM), thereby changing both the abundance of intact polyamines and the substrate available for oxidative catabolism. Intact SPM can scavenge reactive species and chelate Fe^2+^, whereas SMOX- and SSAT–PAOX-mediated oxidation generates H_2_O_2_ and reactive aldehydes. In SRS, severe SMS deficiency produces SPM depletion, SPD accumulation, abnormal catabolism, and mitochondrial and lysosomal stress. In cancer, the consequences of SMS perturbation depend on the residual SPD/SPM profile, oxidase activity, iron availability, and antioxidant capacity, which together influence lipid peroxidation and ferroptosis. The arrows summarize reported relationships and do not imply that every effect is direct or universal.

**Table 1:** Mechanistic evidence linking SMS-dependent polyamine remodeling and related oxidative pathways to cancer phenotypes and redox outcomes.

Type	Evidence Basis	Context	Molecular Pathway	Outcome
Tumor-intrinsic	SMS manipulation	Pancreatic cancer	METTL3–m^6^A–IGF2BP3 → SMS mRNA stability ↑ → SMS/SPM ↑ → AKT–EMT	Growth and metastasis
	SMS manipulation	Colorectal cancer	SMS pathway: SMS ↑ → excessive SPD accumulation ↓ → FOXO3a nuclear translocation ↓ → BIM transcription ↓; MYC pathway: MYC → miR-19a/19b ⊣ BIM	CRC cell survival
Redox	SMS manipulation	HCC: Ferroptosis	SMS-derived SPM → Fe^2+^ chelation → lipid peroxidation ↓	Ferroptosis resistance
	SMOX manipulation	*H. pylori* gastric cancer	SMOX-mediated SPM oxidation → H_2_O_2_ → oxidative DNA damage	Carcinogenesis
	PAOX/SMOX manipulation	Ferroptosis conditions	PAOX/SMOX-mediated polyamine oxidation → H_2_O_2_ → lipid peroxidation ↑	Ferroptosis amplification

Note. Arrows indicate reported directional relationships: ↑ and ↓ indicate increases and decreases, respectively, and ⊣ indicates inhibition.

## Data Availability

The TCGA and GTEx data presented in Supplementary Figure S1 are publicly available and were accessed through GEPIA2 (http://gepia2.cancer-pku.cn/; accessed on 28 July 2026). No new datasets were generated in this study.

## Supplementary Materials

The following supporting information can be downloaded at: https://www.mdpi.com/article/10.3390/antiox15101248/s1; Figure S1: GEPIA2 analysis of *SMS* expression and overall survival; Supplementary Table S1: Selected reported *SMS* variants and clinical features in individuals with Snyder–Robinson syndrome.

## Abbreviations

The following abbreviations are used in this manuscript:

ACC	Adrenocortical carcinoma
AdoMetDC	S-adenosylmethionine decarboxylase
AR	Androgen receptor
AR-FL	Full-length androgen receptor
AR-V7	Androgen receptor splice variant 7
BRCA	Breast invasive carcinoma
CHOL	Cholangiocarcinoma
COAD	Colon adenocarcinoma
CRC	Colorectal cancer
CRPC	Castration-resistant prostate cancer
dcSAM	Decarboxylated S-adenosylmethionine
EMT	Epithelial–mesenchymal transition
ESCA	Esophageal carcinoma
GPX4	Glutathione peroxidase 4
GSH	Glutathione
H_2_O_2_	Hydrogen peroxide
HCC	Hepatocellular carcinoma
HNSC	Head and neck squamous cell carcinoma
ICB	Immune checkpoint blockade
LIHC	Liver hepatocellular carcinoma
LUAD	Lung adenocarcinoma
m^6^A	N^6^-methyladenosine
mTORC1	Mechanistic target of rapamycin complex 1
ODC	Ornithine decarboxylase
OXPHOS	Oxidative phosphorylation
PAH	Pulmonary arterial hypertension
PAOX	N^1^-acetylpolyamine oxidase
PD-L1	Programmed death-ligand 1
PI3K	Phosphoinositide 3-kinase
PRAD	Prostate adenocarcinoma
READ	Rectum adenocarcinoma
ROS	Reactive oxygen species
SAM	S-adenosylmethionine
SMOX	Spermine oxidase
SMS	Spermine synthase
SPD	Spermidine
SPM	Spermine
SRM	Spermidine synthase
SRS	Snyder–Robinson syndrome
SSAT	Spermidine/spermine N^1^-acetyltransferase
STAD	Stomach adenocarcinoma
TPM	Transcripts per million
Treg	Regulatory T cell
UCEC	Uterine corpus endometrial carcinoma
