# Supplementary material for: Spermine Synthase at the Polyamine–Redox Interface: Mechanistic Evidence from Snyder–Robinson Syndrome and Cancer
Source: Antioxidants (Basel). 2026 Sep 28;15(10):1248. doi: 10.3390/antiox15101248 (PMC13700777; doi:10.3390/antiox15101248)
Supplement: Supplementary file 1 [file antioxidants-15-01248-s001.zip › Table S1.pdf]

Table S1. Selected reported *SMS* variants and clinical features in individuals with Snyder–Robinson syndrome.

cDNA variant	Protein consequence	Core clinical features	Additional reported features	Reference
c.166G>A	p.Gly56Ser	Severe intellectual disability, epilepsy, hypotonia, facial dysmorphism	Skeletal abnormalities, high myopia, pectus carinatum	[32]
c.174T>A	p.Phe58Leu	Developmental delay, seizures	Severe osteoporosis, scoliosis	[33]
c.200G>A	p.Gly67Glu	Intellectual disability, early-onset epilepsy, facial dysmorphism	Ectopic right kidney, nasal speech, thin habitus	[34]
c.329+5G>A	Aberrant splicing with premature protein truncation	Mild-to-moderate intellectual disability, hypotonia, cerebellar dysfunction	Thin habitus, facial asymmetry	[28]
c.334C>G	p.Pro112Ala	Hypotonia, motor delay, epilepsy	Diffuse osteopenia, low-energy humeral fracture; no vertebral fracture or scoliosis	[35]
c.335C>T	p.Pro112Leu	Mild intellectual disability, seizures, gait abnormalities	Recurrent fractures, severe scoliosis, severe muscle atrophy	[36]
c.388C>T	p.Arg130Cys	Developmental and epileptic encephalopathy, hypotonia, facial dysmorphism	Congenital heart disease, hyperinsulinemic hypoglycemia, hypothyroidism, bilateral sensorineural hearing loss	[30,37]
c.395T>G	p.Val132Gly	Intellectual disability, asthenic habitus, osteoporosis	Prominent lower lip, high-arched or cleft palate, long, slender fingers and toes	[38]
c.443A>G	p.Gln148Arg	Intellectual disability, seizures, osteoporosis	Recurrent respiratory infections, facial dysmorphism	[29]
c.746A>G	p.Tyr249Cys	Learning disability, epilepsy, hypotonia, osteoporosis	Absence of intellectual disability, recurrent fractures, truncal weakness	[31]

c.831G>T	p.Leu277Phe	Intellectual disability, gait abnormalities, multiple fractures, seizures	Prolonged seizure activity complicated by fatal hypoxic–ischemic encephalopathy	[39]
c.905C>T	p.Ser302Leu	Intellectual disability, seizures, speech impairment	Reduced bone density, limited ambulation	[40]
c.908_911del	p.Met303LysfsTer3	Severe growth retardation, dysmorphism, neurologic and feeding difficulties	Hirschsprung disease, persistent thrombocytopenia and anemia, hepatic abnormalities, renal tubulopathy	[41]
c.983A>G	p.Tyr328Cys	Mild intellectual disability, developmental delay, slender habitus	No facial asymmetry, short stature, unsteady gait	[42]

All cDNA variants are numbered according to the *SMS* reference transcript NM_004595.5, and protein consequences are based on NP_004586.2 where applicable. Clinical features are those reported in the cited individual(s) and should not be interpreted as established variant-specific genotype–phenotype associations.
